# Multi-country assessment of residual bio-efficacy of insecticides used for indoor residual spraying in malaria control on different surface types: results from program monitoring in 17 PMI/USAID-supported IRS countries

**DOI:** 10.1186/s13071-017-2608-4

**Published:** 2018-01-30

**Authors:** Dereje Dengela, Aklilu Seyoum, Bradford Lucas, Benjamin Johns, Kristen George, Allison Belemvire, Angela Caranci, Laura C. Norris, Christen M. Fornadel

**Affiliations:** 1 U.S. PMI Africa Indoor Residual Spraying Project, Abt Associates, 4550 Montgomery Ave, Suite 800 N, Bethesda, MD 20814 USA; 2 U.S. President’s Malaria Initiative USAID, 1300 Pennsylvania Avenue NW, Washington, DC 20523 USA

**Keywords:** Malaria, IRS, Insecticide resistance, PMI, USAID, Residual bioassay, Alpha-cypermethrin, Bendiocarb, Deltamethrin, Pirimiphos-methyl CS

## Abstract

**Background:**

Indoor residual spraying (IRS) is the application of insecticide to the interior walls of household structures that often serve as resting sites for mosquito vectors of malaria. Human exposure to malaria vectors is reduced when IRS involves proper application of pre-determined concentrations of the active ingredient specific to the insecticide formulation of choice. The impact of IRS can be affected by the dosage of insecticide, spray coverage, vector behavior, vector susceptibility to insecticides, and the residual efficacy of the insecticide applied. This report compiles data on the residual efficacy of insecticides used in IRS campaigns implemented by the United States President’s Malaria Initiative (PMI)/United States Agency for International Development (USAID) in 17 African countries and compares observed length of efficacy to ranges proposed in World Health Organization (WHO) guidelines. Additionally, this study provides initial analysis on variation of mosquito mortality depending on the surface material of sprayed structures, country spray program, year of implementation, source of tested mosquitoes, and type of insecticide.

**Methods:**

Residual efficacy of the insecticides used for PMI/USAID-supported IRS campaigns was measured in Benin, Burkina Faso, Ethiopia, Ghana, Kenya, Liberia, Madagascar, Malawi, Mali, Mozambique, Nigeria, Rwanda, Senegal, Tanzania, Uganda, Zambia and Zimbabwe. The WHO cone bioassay tests were used to assess the mortality rate of mosquitoes exposed to insecticide-treated mud, wood, cement, and other commonly used housing materials. Baseline tests were performed within weeks of IRS application and follow-up tests were continued until the mortality of exposed mosquitoes dropped below 80% or the program monitoring period ended. Residual efficacy in months was then evaluated with respect to WHO guidelines that provide suggested ranges of residual efficacy for insecticide formulations recommended for use in IRS. Where the data allowed, direct comparisons of mosquito mortality rates were then made to determine any significant differences when comparing insecticide formulation, country, year, surface type, and the source of the mosquitoes used in testing.

**Results:**

The residual efficacy of alpha-cypermethrin ranged from 4 to 10 months (average = 6.4 months), with no reported incidents of underperformance when compared to the efficacy range provided in WHO guidelines. Deltamethrin residual efficacy results reported a range of 1 to 10 months (average = 4.9 months), with two instances of underperformance. The residual efficacy of bendiocarb ranged from 2 weeks to 7 months (average = 2.8 months) and failed to achieve proposed minimum efficacy on 14 occasions. Lastly, long-lasting pirimiphos-methyl efficacy ranged from 2 months to 9 months (average = 5.3 months), but reported 13 incidents of underperformance.

**Conclusions:**

Much of the data used to determine application rate and expected efficacy of insecticides approved for use in IRS programs are collected in controlled laboratory or pilot field studies. However, the generalizability of the results obtained under controlled conditions are limited and unlikely to account for variation in locally sourced housing materials, climate, and the myriad other factors that may influence the bio-efficacy of insecticides. Here, data are presented that confirm the variation in residual efficacy observed when monitoring household surfaces sprayed during PMI/USAID-supported IRS campaigns. All insecticides except alpha-cypermethrin showed evidence of failing to meet the minimum range of residual efficacy proposed in WHO criteria at least once. However, this initial effort in characterizing program-wide insecticide bio-efficacy indicates that some insecticides, such as bendiocarb and pirimiphos-methyl, may be vulnerable to variations in the local environment. Additionally, the comparative analysis performed in this study provides evidence that mosquito mortality rates differ with respect to factors including: the types of insecticide sprayed, surface material, geographical location, year of spraying, and tested mosquitoes. It is, therefore, important to locally assess the residual efficacy of insecticides on various surfaces to inform IRS programming.

**Electronic supplementary material:**

The online version of this article (10.1186/s13071-017-2608-4) contains supplementary material, which is available to authorized users.

## Background

Today, the two principal methods used for malaria vector control worldwide are indoor residual spraying (IRS) and long-lasting insecticidal nets [1, 2]. Historically, IRS has been the primary intervention method in vector control efforts [1, 2]. Since its introduction as a vector control tool in 1945, IRS has proven successful in reducing the prevalence and incidence of malaria by reducing the level of transmission through killing or repelling malaria vectors [3, 4]. In 1969, the global malaria strategy shifted from a time-limited eradication to a long-term control program, resulting in a decline in the number of countries implementing IRS as a primary mechanism for vector control. However, several countries continued IRS in some capacity after the eradication era, including: Equatorial Guinea (Bioko Island), Eritrea, Ethiopia, Mozambique, South Africa, Swaziland and Zimbabwe [5, 6].

Since 2006, several countries in sub-Saharan Africa have introduced or re-started or expanded the reach of IRS with financial and technical support primarily from the United States President’s Malaria Initiative (PMI)/United States Agency for International Development (USAID) and the Global Fund for AIDS, Tuberculosis and Malaria as part of renewed global commitment to control and ultimately eliminate malaria [1, 7]. According to the 2012 World Malaria Report, the proportion of people at risk of malaria protected by IRS increased from less than 5% in 2005 to 11% in 2010 in Africa [8]. However, spray coverage declined to 8% in 2012, 7% in 2013 and 6% in 2014 [9, 10]. The cost associated with switching from pyrethroid insecticides to alternatives such as carbamates and/or organophosphates led to a decrease in the proportion of people protected by IRS [9]. These switches were, in turn, prompted by the emergence and spread of vector resistance to pyrethroids, to DDT, and, in some countries, to carbamates. The alternative classes of insecticides are much more expensive to purchase and generally have a shorter residual life than pyrethroids (the exception being the capsule suspension formulation of the organophosphate insecticide pirimiphos-methyl, which has a longer residual life) [11]. This, in turn, requires repeated application of carbamates or organophosphates (other than pirimiphos-methyl capsule suspension) in areas of perennial malaria transmission in order to provide the same level of protection as pyrethroids [12–14].

The effectiveness of IRS depends on several factors, including: vector resting habit (effective when the vector is endophilic), quality of spraying, spray coverage, susceptibility of the local vector(s) to the insecticide used for IRS [15], and the residual efficacy of the sprayed insecticide [16, 17]. The residual efficacy and persistence of insecticide are known to be affected by several factors that include, but are not limited to, the nature of the sprayed surfaces (mud, wood, cement, thatched, etc.) [17–21], pH of the sprayed substrates [22], physicochemical properties of the sprayed insecticide (vapor pressure and volatility) [23], availability of degrading bacteria [20], insecticide formulation [wettable powder (WP), capsule suspension (CS), emulsifiable concentrate (EC), suspension concentrate (SC) or wettable granules (WG)] [7, 24, 25], the amount of insecticide deposited on sprayed surfaces [26], and temperature and humidity [19, 27].

PMI’s contribution to IRS coverage in Africa is substantial (Table 1). From 2010 to 2014, PMI protected between 18.2 million and 30.3 million people with IRS each year, covering between 35% and 52% of the total population protected by IRS in Africa [1, 8, 26, 28, 29–31]. Entomological monitoring is an integral part of the PMI-supported IRS program. The World Health Organization’s (WHO’s) standard cone bioassay is regularly used to assess the quality of spraying, and subsequently, to monitor the residual life of the sprayed insecticides. These data are used to guide the insecticide selection process and to determine appropriate timing and intervals for spraying in order to maximize the impact of IRS. The majority of insecticide residual efficacy studies have been conducted either in the laboratory, using experimental huts, or in residential houses under controlled conditions in the field [7, 22, 26, 32]. In the controlled studies, spray operators were purposefully and specifically recruited and trained for the studies. Spraying was also conducted under the direct supervision of the investigators. Results from these studies may not reflect what occurs during large-scale IRS implementation, which deploys large groups of spray operators and does not necessarily have trained supervisors individually overseeing the spraying of every house. Given the complexity of an IRS program, implementation is likely imperfect even when the operation is implemented following best practices and using state of the art spraying techniques. Additionally, the composition of sprayed surfaces encountered during campaigns varies by location and between years, which may impact residual efficacy.

**Table 1:** IRS coverage in Africa

Year	Total population protected by IRS in Africa	Total population protected through PMI support in Africa	% population protected with PMI support
2010	78 million	27,199,063	35
2011	77 million	28,344,173	36
2012	58 million	30,297,000	52
2013	55 million	21,801,615	40
2014	50 million	18, 270,723	37

In order to assess the residual efficacy of insecticides used in a programmatic setting, we present data from 17 countries collected as part of PMI/USAID-supported IRS program monitoring and compare observed lengths of residual efficacy to expected duration of action put forth by the WHO. This comparison was performed for five programmatically approved and implemented insecticides: alpha-cypermethrin WP, deltamethrin WG, bendiocarb WP, pirimiphos-methyl CS and pirimiphos-methyl EC. We also evaluate the impact of locality, type of surface sprayed, program year, type of insecticide, and source of test specimens on overall mosquito mortality. The results demonstrate the importance of locally generated residual activity data to inform IRS programming, and they can help inform discussions about when and where to use IRS and associated insecticides.

## Methods

### Study areas

Insecticide residual efficacy monitoring was conducted in 17 PMI/USAID-supported IRS countries: Benin, Burkina Faso, Ethiopia, Ghana, Kenya, Liberia, Madagascar, Malawi, Mali, Mozambique, Nigeria, Rwanda, Senegal, Tanzania, Uganda, Zambia and Zimbabwe. Residential houses sprayed with one of the five insecticides/formulations (alpha-cypermethrin WP, deltamethrin WG, bendiocarb WP and pirimiphos-methyl CS/EC) were monitored for the residual life of insecticides from 2008 to 2015.

Within each country, specific sites were selected for insecticide residual efficacy monitoring. Selection criteria took into consideration factors such as accessibility, distance from the insectary, feasibility for transporting susceptible colonies of mosquitoes for testing, and areas sprayed with PMI support. Within about a week of the start of an IRS campaign, entomological monitoring teams obtained lists of sprayed and unsprayed houses from the spray record. Test houses were randomly selected from the list of houses sprayed within the sites selected for monitoring. Either the test houses themselves or unsprayed houses closest to the test houses were used for control tests. In the event that the sprayed houses were used for both exposure and control tests, the wall surfaces were covered with cardboard for control tests to avoid insecticide contact. Household structure materials were observed during the first tests and effort was made to include representative housing materials at each site. The walls of the houses were most commonly made of mud, wood, or cement; however, additional housing materials were also noted, including: plaster with dung, mud coated with cement, mud coated with kaolin, thatch, bamboo, brick, paper and painted surfaces (Additional file 1: Table S1). In the absence of houses with wood walls, mosquitoes were exposed to doors and windows (which were made of wood) to capture the performance of insecticides on wood surfaces for comparison.

### Spraying and dosages

IRS was implemented in accordance with WHO [33] and PMI best practices [34] to ensure a high quality of spraying and the safety of the residents, spray operators and environment. PMI’s IRS implementing partners (Abt Associates, Chemonics International and RTI International) carried out the spraying on an annual basis with the exception of Rwanda. In Rwanda, two rounds of spraying per year were conducted in 2015 owing to the short residual life of the insecticide used (bendiocarb) in order to cover two transmission peaks during the year. Spray operators used Hudson X-Pert or Goizper hand compression sprayers fitted with flat fan nozzles to spray the interior walls and non-metal ceilings of eligible structures in IRS-targeted areas. All spray operators, team leaders and spray supervisors were trained prior to spray operations. Spray operators were provided with personal protective equipment including: long-sleeved gloves, mouth masks, face shields, coveralls, rubber boots and neck covers to prevent exposure to insecticide. Trained mobilizers and mass media communications informed residents in advance of the date, implementation and importance of spraying, including explanations of residents’ role before, during, and after spraying.

Spray operators mixed pre-packed sachets or bottles of insecticide with 10 or 8 l of water to obtain the recommended suspension of insecticide and sprayed to cover 250 or 200 square meters of sprayable surfaces, respectively. Deltamethrin WG, alpha-cypermethrin WP, bendiocarb WP and pirimiphos-methyl CS/EC were sprayed at dosages of 0.025 g a.i./m^2^, 0.03 g a.i./m^2^, 0.4 g a.i./m^2^ and 1 g a.i./m^2^, respectively, as recommended by the World Health Organization Pesticide Evaluation Scheme (WHOPES) [35, 36]. Spray operations staff informed residents to stay out of the structure for at least two hours until the sprayed surfaces were dried.

### Bioassays

Bioassays were performed using the WHO standard cone bioassay test procedures [37]. Most of the countries have local capacity to collect high quality cone bioassay data and functional insectaries with established, susceptible mosquito colonies. The PMI project supported training efforts, and an experienced entomologist provided support during the field data collection in countries with limited human resource capacity.

One- to five-day-old female mosquitoes were used for testing. Mosquitoes included wild female *Anopheles gambiae* (*s.l.*) reared from field-collected larvae and pupae and *Anopheles gambiae* Kisumu strain or an *Anopheles arabiensis* susceptible strain reared in an insectary. Insecticide residual activity data collected using wild mosquito populations from areas of known or suspected resistance were not included in this report. The susceptible mosquito colonies used for the tests in Benin, Burkina Faso, Ghana, Kenya, Malawi, Mali, Nigeria, Rwanda, Senegal, Tanzania, Uganda, Zambia and Zimbabwe were *Anopheles gambiae* (*s.s.*) Kisumu strains. The susceptible mosquitoes in Ethiopia and Mozambique were *Anopheles arabiensis*.

In countries without access to a susceptible mosquito colony, tests were done with locally collected, wild mosquitoes. Susceptibility of the wild mosquitoes to the applicable insecticides sprayed was confirmed using a WHO standard susceptibility test [38] before mosquitoes from the same population were used for cone bioassay tests. Only wild *Anopheles gambiae* (*s.l.*) were used for the testing in Liberia, Madagascar, Mali (2012) and Uganda (2010). In these instances, larvae collected from breeding sites were collected and reared to adults, such that the F0 generations were used in both cone bioassays and susceptibility tests as per the WHO protocol [37]. Occasionally, wild mosquitoes were used for cone bioassay testing in parallel with the susceptible colony in order to compare field mosquito mortality with susceptible colony mortality. Benin (2014), Ethiopia (2014 and 2015), Ghana (2013, 2014 and 2015) and Zambia (2015) simultaneously used wild and susceptible-colony mosquitoes for cone bioassay tests.

The data were collected as part of regular program monitoring, which started one week after the start of spraying. One week was chosen (i) to allow a sufficient number of houses to be sprayed to provide an adequate sample size for the study and (ii) to give IRS program managers the opportunity to take corrective action if poor quality spraying was suspected at the beginning of the spraying operation. Although all efforts were made to conduct the first cone bioassays within seven days of the start of spraying, baseline data for some campaigns were captured one month after spraying began. In most countries, subsequent bioassays were carried out monthly. However, at times, a few countries had to conduct residual activity monitoring at wider intervals due to an insufficient number of mosquitoes.

In most PMI-supported countries, entomological monitoring, including residual bio-efficacy of insecticides, was planned for a period of about six months that overlap with the main malaria transmission season. In most cases, monitoring was stopped when either mosquito mortality dropped significantly or at the end of the six months, irrespective of the efficacy of the insecticides. Monitoring continued in some countries for a few months after cone bioassay test mortality dropped below 80%. Since 2012, PMI-supported programs have encouraged systematic collection of cone bioassay data until mosquito test mortality drops below the 80% efficacy threshold used by WHO (Table 2) [39]. However, a few countries [Ethiopia (2014), Ghana (2012), Madagascar (2012 and 2013), Uganda (2012) and Tanzania (2012)] have failed to comply with this standard approach, and they were forced to stop data collection before the test mortality dropped below the threshold (Additional file 2: Table S2, Additional file 3: Table S3, Additional file 4: Table S4) because of a lack of mosquitoes needed for the tests.

When collecting the cone bioassay data, staff used either masking tape or nails to diagonally fix exposure chambers to the sprayed surfaces at three different heights: ‘lower’ (0.5 m above the ground), ‘middle’ (1.0 m) and ‘top’ (1.5 m). However, in Ghana, all three cones were placed at the same level (1.0 m above the ground). When windows or doors were used to represent wooden surfaces, only one cone was fixed. Ten to 15 non-blood-fed female mosquitoes were introduced to each chamber and exposed to the surface for 30 min. Unsprayed rooms or insecticide-free cardboard placed on sprayed surfaces served as a control. At the end of the exposure time, staff counted the number of knocked-down mosquitoes and transferred the mosquitoes to insecticide-free paper cups covered with netting and supplied with a 10% sugar solution. The cups were placed in a wooden box, or cool box, which was covered with a damp towel to keep an optimum microclimate for the mosquitoes. Staff then assessed mortality after a 24 h holding period, and calculated the percent mortality. When control mortality was between 5% and 20%, exposure mortality was corrected using Abbott’s formula [40]. All tests with control mortality greater than 20% were discarded and the tests were repeated or data excluded from this paper. Cone bioassay test results are presented both in tables included as additional files and graphs. Only cone bioassay results conducted on the main surface types (mud, cement and wood) are included in the graphs.

### Data analysis

For comparison of the length of residual efficacy observed in the field against WHO criteria, the number of months during which mosquito mortality remained greater than or equal to 80% was noted. Descriptive statistics were calculated in an effort to describe basic measures of programmatic residual efficacy for each insecticide.

To determine the residual efficacy of the sprayed insecticides, we analyzed test mortality rates at each time point and interpreted results according to WHO protocols [39]. The average test mortality by surface type was calculated for each year and country. The sprayed insecticide was considered effective when mosquito test mortality rates were greater than 80% [39]. When test mortality oscillated above and below 80%, the last point when it dropped below 80% was considered effective residual activity for that insecticide. Multiple Poisson regression was used to compare the performance of insecticides between different substrates (within a country), assess whether there was any change in adjusted test mortality over time in bioassay tests (within a country), and compare the same insecticide used in two different countries controlling for surface type. For these comparisons, bioassay data collected throughout the monitoring period, including those collected after test mortality dropped below 80%, was incorporated. The outcome variable for the regression was the number of dead mosquitoes after a 24 h holding period per test (sum of all replicate of exposures) per house, with the number of mosquitoes tested included as an exposure variable. Other variables, such as wall surface, time since insecticide application, and year, were included in the regression as categorical variables when relevant for the comparison being made; we also included fixed effects for country and year as appropriate to account for potential autocorrelation. The regressions were limited to include only data relevant to the comparison being made (e.g. when comparing mud and dung walls, data from mosquitoes exposed to cement were excluded from the regression).

We did detailed comparative analyses of residual efficacies on different surface types and periods for 13 selected countries: Benin, Ethiopia, Ghana, Liberia, Madagascar, Mali, Mozambique, Nigeria, Rwanda, Senegal, Zambia and Zimbabwe. Presence of readily available cone bioassay data by replicate was used as selection criteria to include countries for comparisons using statistical data analysis. A *P*-value of < 0.05 was considered significant. All statistical tests were done using StataMP 12.1, and comparisons were made using the Wald (Z) tests in Poisson regressions.

## Results

### Observed residual efficacy of IRS insecticides from bioassays

**Fig. 1:**
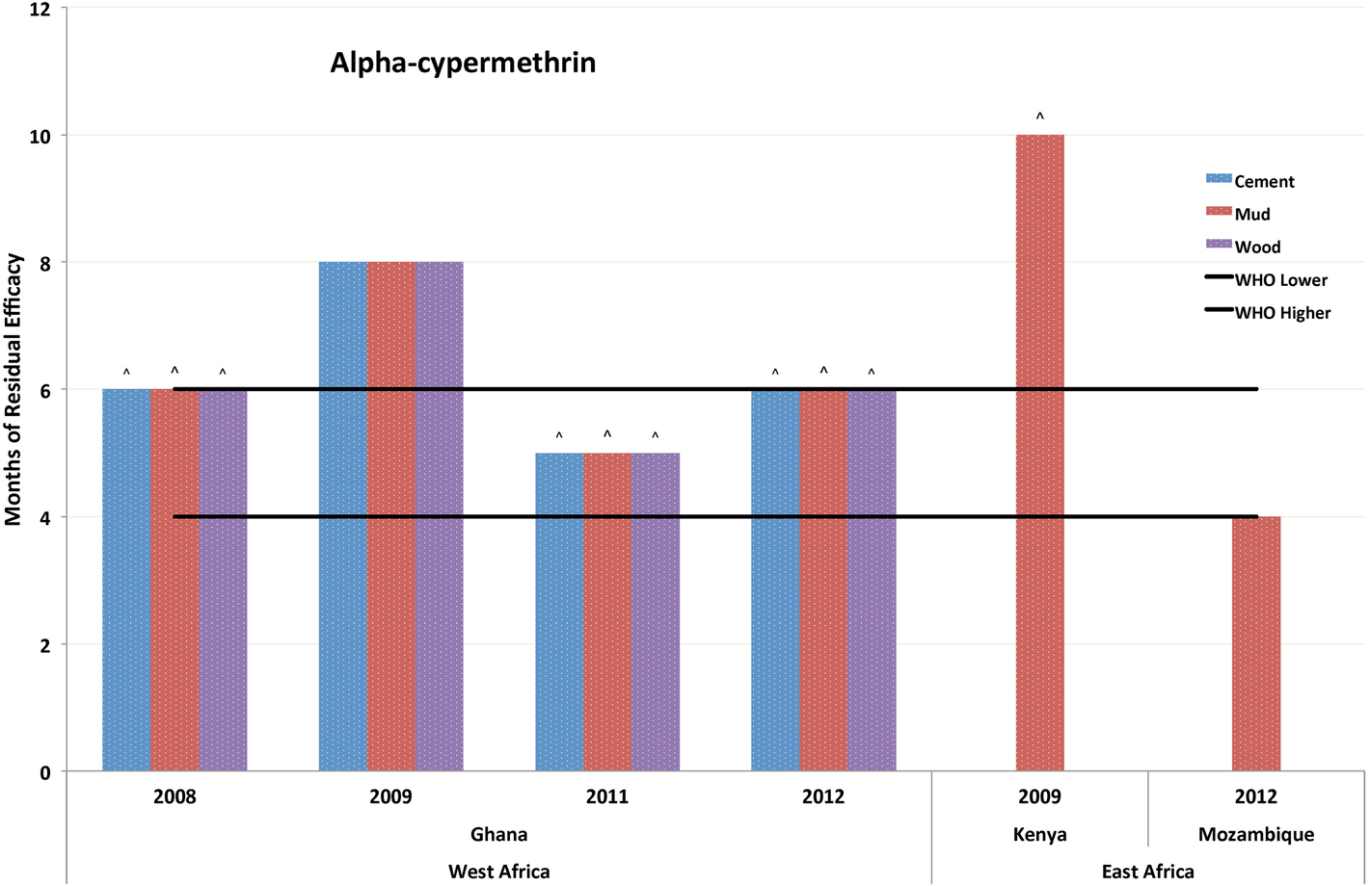
Residual bio-efficacy of alpha-cypermethrin sprayed on different surface types measured using WHO cone bioassays 2008–2012 in Ghana, Kenya and Mozambique. ^: data label designates instances where testing was ended before mosquito mortality fell below the WHO mortality cut-off of 80%

**Fig. 2:**
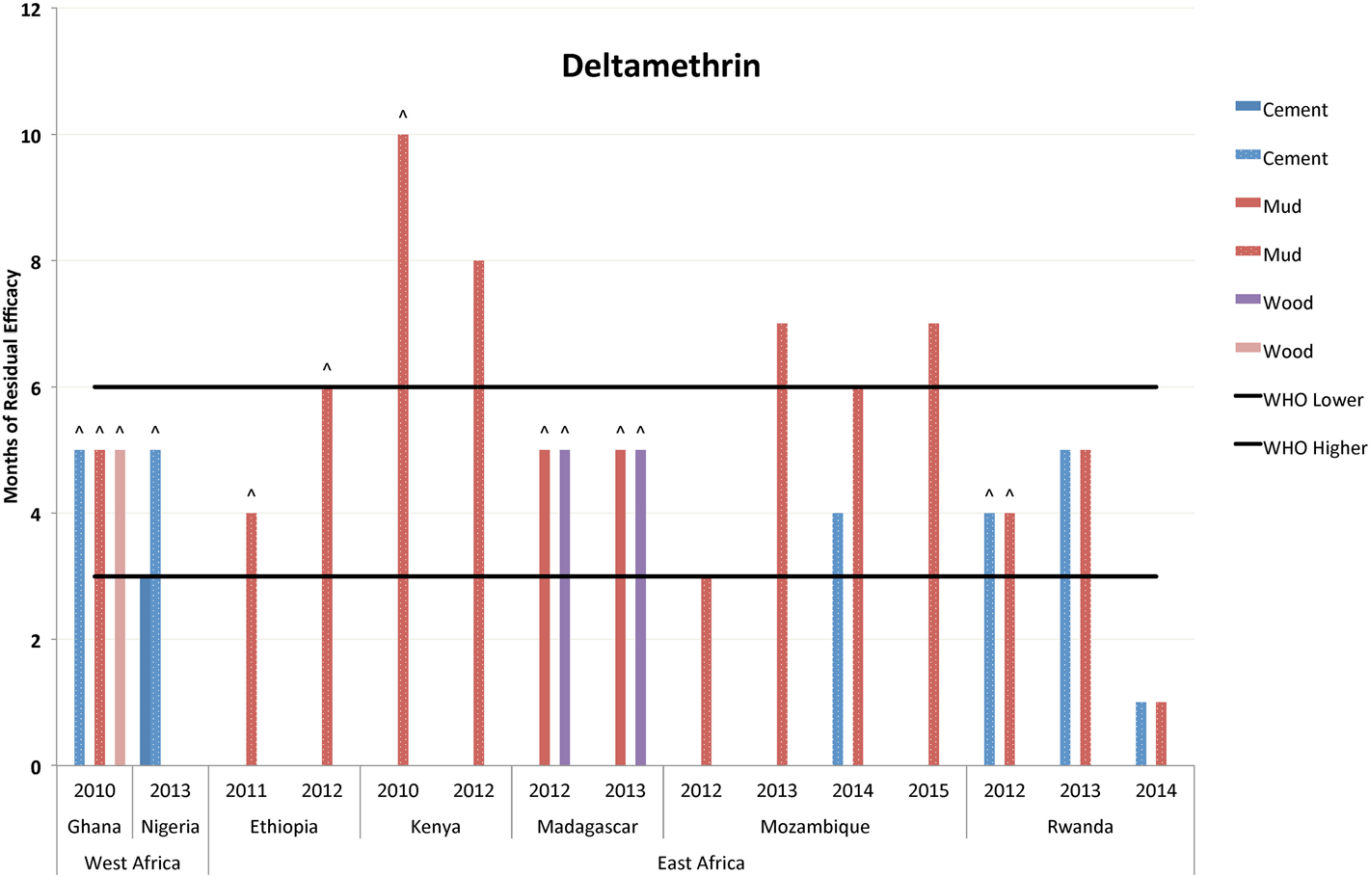
Residual bio-efficacy of deltamethrin sprayed on different surface types measured using WHO cone bioassays 2010–2015 in Africa. ^: data label designates instances where testing was ended before mosquito mortality fell below the WHO mortality cut-off of 80%

**Fig. 3:**
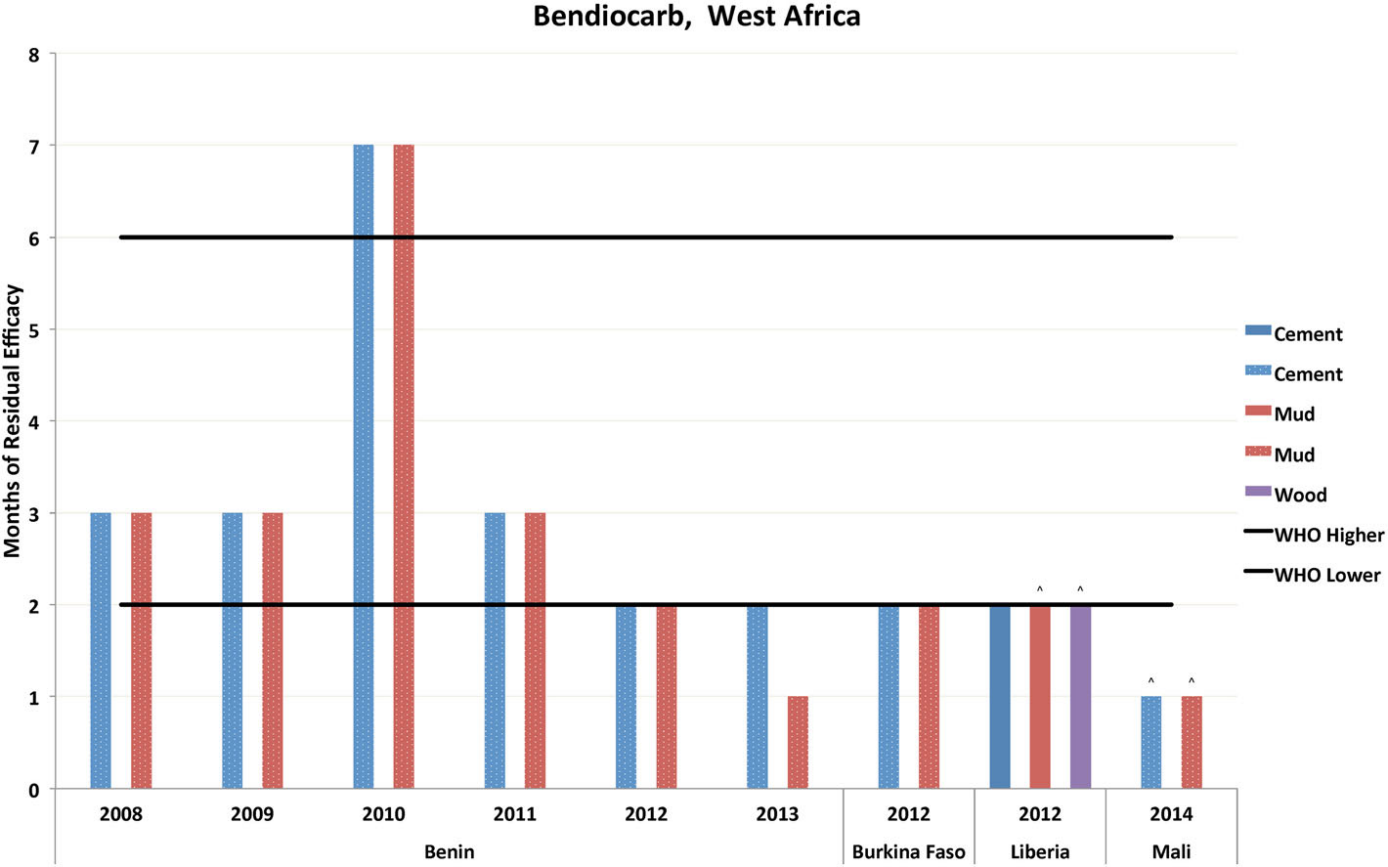
Residual bio-efficacy of bendiocarb sprayed on different surface types measured using WHO cone bioassays 2010–2015 in East Africa. ^: data label designates instances where testing was ended before mosquito mortality fell below the WHO mortality cut-off of 80%

**Fig. 4:**
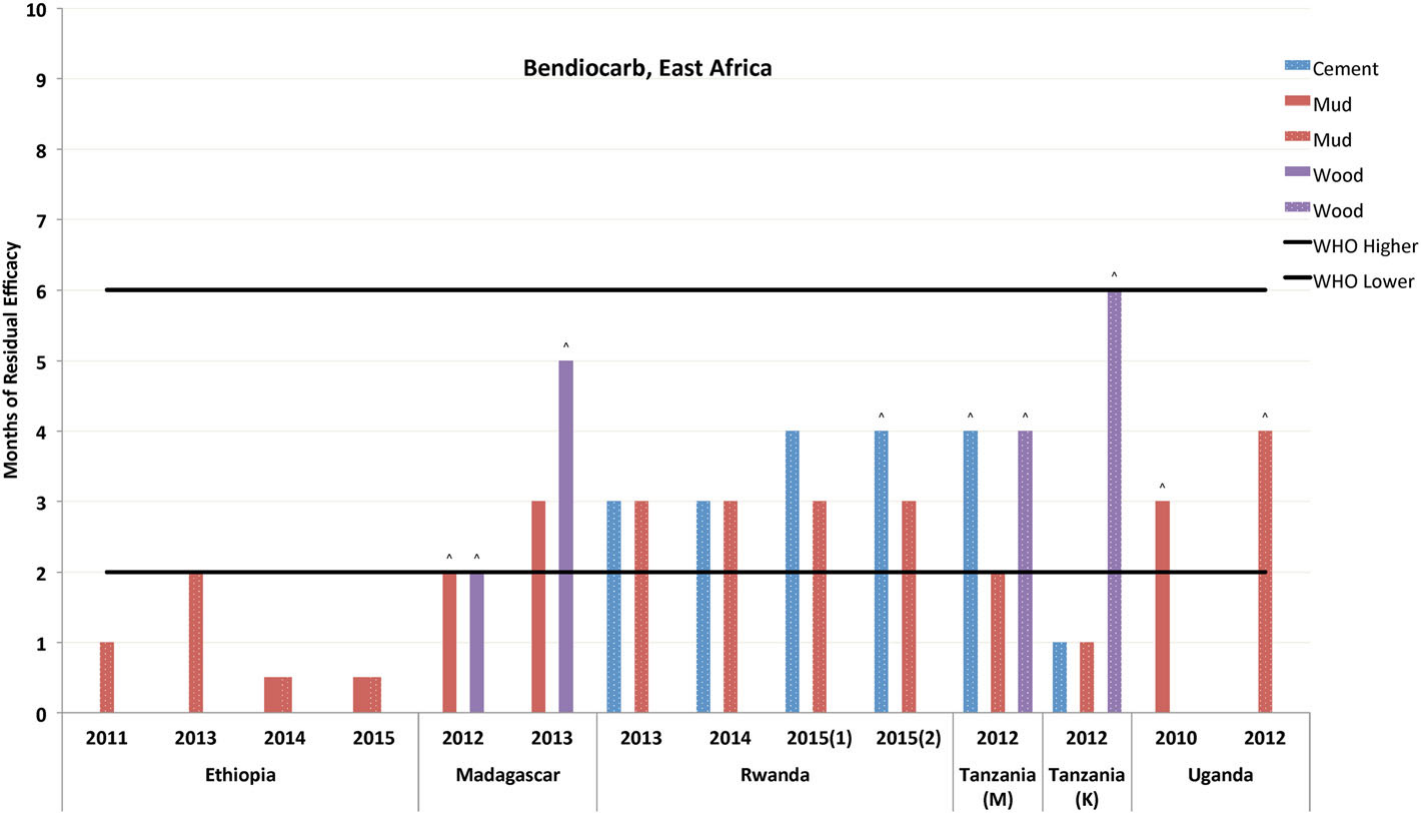
Residual bio-efficacy of bendiocarb sprayed on different surface types measured using WHO cone bioassays 2008–2014 in West Africa. ^: data label designates instances where testing was ended before mosquito mortality fell below the WHO mortality cut-off of 80%

**Fig. 5:**
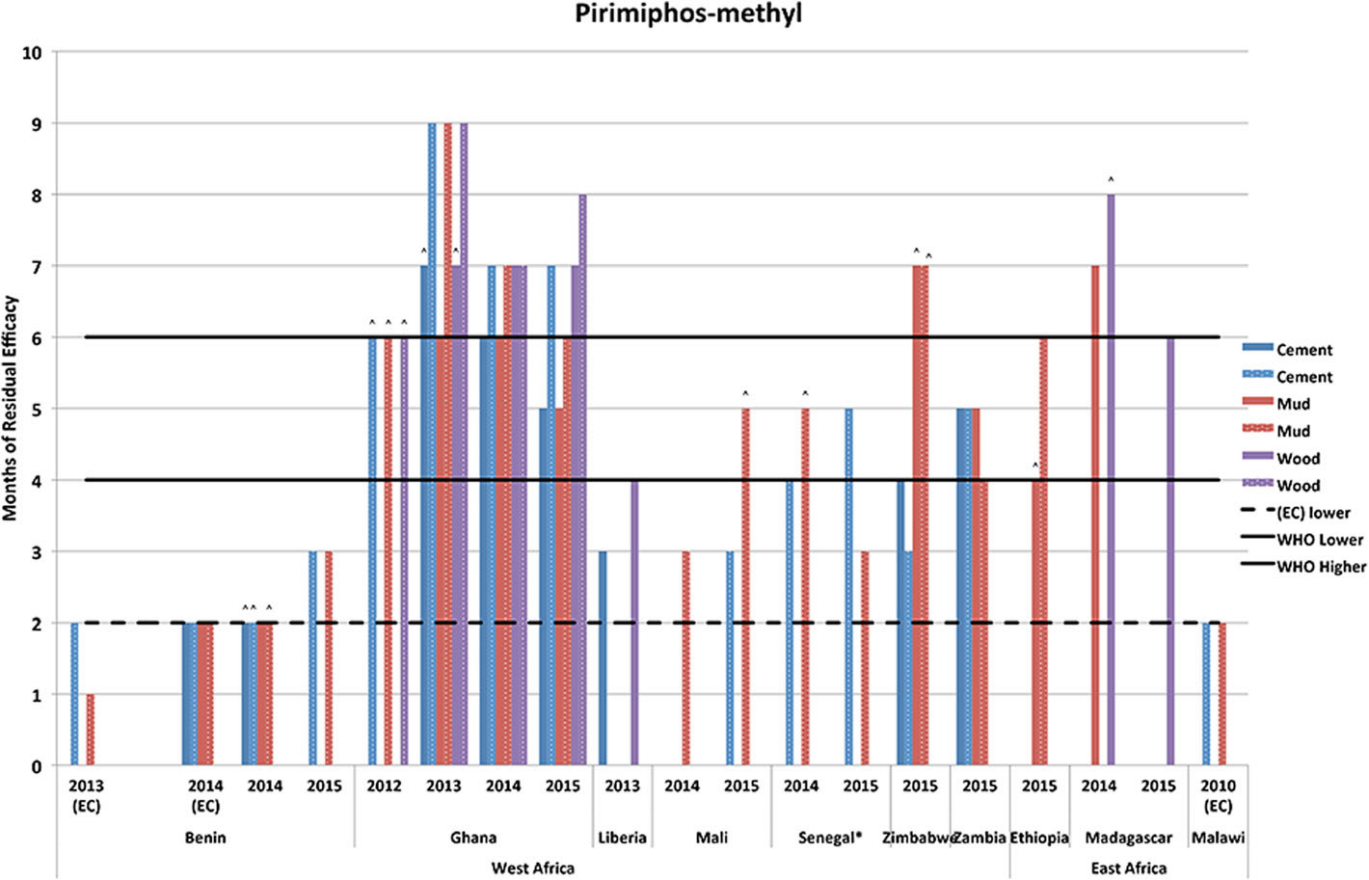
Residual bio-efficacy of pirimiphos-methyl sprayed on different surface types measured using WHO cone bioassays 2010–2015 in East and West Africa. ^: data label designates instances where testing was ended before mosquito mortality fell below the WHO mortality cut-off of 80%

The observed time when mosquito mortality rates first dropped below 80% or when residual monitoring activities were stopped, even if mortality was more than 80%, are presented in comparison to WHO guidelines for upper and lower limits of residual efficacy (in months) (Table 2, Figs. 1, 2, 3, 4, 5).

**Table 2:** Residual bio-efficacy in months of insecticides from routine program monitoring, WHO guidelines, and previous studies

Insecticide	Range of residual efficacy from program monitoring^a^	Suggested residual efficacy per WHO report^b^	Residual efficacy found in other studies^c^
Alpha-cypermethrin WP	4–10 (*n* = 14)	4–6	• Up to 14 (plastic coated plywood), < 1 (cement) in Sao Tome and Principe [41]; • 1.5 against sand flies in Morocco [42]^d^; • 2.75 to 4 in India [43]^e^
Deltamethrin WG	1–10 (*n* = 24)	3–6	• 6.5 (concrete), 5 (mud) and 3.8 (wood) in Cameroon [18]; • 3 (plastered), 2.5 (mud) and 1 (cement) in Iran [26]^f^; • 15 months (wood, bamboo and brick) in Malaysia [45]^g^
Bendiocarb WP	< 1–7 (*n* = 59)	2–6	• 1.5 (red clay and mixture of red clay and cement) and 1.75 (mixture of sand and cement) in Benin [21]; • Between 2 and 5 months in Equatorial Guinea [46]; • 6 in Mozambique [47]; • > 3.25 in Cameroon [18]
Pirimiphos-methyl CS	2–9 (*n* = 57)	4–6	• 9 months in Benin [51]^h^; • 8 (mud, cement plastered, lime wash, water paint) and > 9 (oil paint) in Tanzania [54]; • 4 (mud) and 5 (concrete) in Côte d’Ivoire [11]
Pirimiphos-methyl EC	2 (*n* = 8)	2–3	na

^a^Source: Authors’ calculations from field collected data

^b^Sources: [32, 34]

^c^Sources as listed. (*n* = the number of test observations included in the current dataset)

^d^For alpha-cypermethrin SC;

^e^alpha-cypermethrin WP and WG; doses of 20 mg/m^2^ and 30 mg/ m^2^; no significant difference was observed between the two formulations and dosages

^f^50, 40 and 25 mg/m^2^ dosages, respectively; tested on laboratory-reared *Anopheles stephensi* strain

^g^*Aedes aegypti* and *Aedes albopictus* strains

^h^Dosage of 0.5 g/m^2^

#### Alpha-cypermethrin (WP)

Residual efficacy testing was performed in conjunction with spray campaigns that took place in 2008, 2009, 2011 and 2012. During those years, the range (in months) of residual efficacy of alpha-cypermethrin spanned from four months to greater than 10 months (Fig. 1 and Additional file 2: Table S2). Cement, mud and wood surfaces were all included in bioassays performed in Ghana, Kenya and Mozambique. The average length of observed efficacy was 6.36 months (± 1.60). Of the 14 total testing observations, there were no reported instances where the length of residual efficacy did not meet the minimum proposed in WHO guidelines (range of 4–6 months). There were four instances where continued efficacy monitoring showed that alpha-cypermethrin consistently resulted in greater than 80% mosquito mortality for longer than six months.

#### Deltamethrin (WG)

For deltamethrin, residual efficacy testing was performed in conjunction with spray campaigns that took place from 2010 to 2015. The range (in months) of residual efficacy spanned from one month to more than 10 months (Fig. 2 and Additional file 3: Table S3). Cement, mud and wood surfaces were all included in bioassays performed in Ethiopia, Ghana, Kenya, Madagascar, Mozambique, Nigeria and Rwanda. The average length of observed efficacy was 4.92 months (± 1.95). Of the 24 total testing observations, there were two reported instances (8.33%) where the length of residual efficacy did not meet the minimum proposed in WHO guidelines (range of 3–6 months). The two observations were from the same country and year, Rwanda in 2014. There were also four instances where continued efficacy monitoring showed that deltamethrin resulted in greater than 80% mosquito mortality for longer than six months.

#### Bendiocarb (WP)

For bendiocarb, residual efficacy testing was performed in conjunction with spray campaigns that took place from 2008 to 2015. The range (in months) of residual efficacy spanned from less than two weeks to seven months. Cement, mud, mud covered with cement, wood, dung, painted, and brick surfaces were all included in bioassays performed in Benin, Burkina Faso, Ethiopia, Liberia, Madagascar, Mali, Rwanda, Tanzania and Uganda. The average length of observed efficacy was 2.79 months (± 1.65). Of the 59 testing observations, there were 14 observed instances (23.73%) where the length of residual efficacy did not meet the minimum proposed in WHO guidelines (range of 2–6 months). Of these 14 observations, eight were observed failures on mud, three on mud covered with cement, two on cement, and one on painted surfaces. Of those eight observations that fell short of being in the WHO range on mud surfaces, five were reported from Ethiopia alone. The three failures on mud coated with cement were reported from Mali. There were only two instances where continued efficacy monitoring showed that bendiocarb consistently resulted in greater than 80% mosquito mortality for longer than six months.

Figures 3 and 4 and Additional file 4: Table S4 represent the residual efficacy of bendiocarb on cement, mud, and wood surfaces, which represented 32% of the total observations listed in Additional file 1: Table S1. The table was broken down by region of Africa due to the large amount of data collected. The results presented in Fig. 3 summarize observations from countries in West Africa (Benin, Burkina Faso, Liberia and Mali) and in this region the average residual efficacy was 2.43 months (± 1.68). The data presented in Fig. 4 are representative of observations from countries in East Africa (Ethiopia, Madagascar, Rwanda, Tanzania and Uganda). Here, the average length of residual efficacy, 3.0 months (± 1.61), was slightly longer than that observed in West Africa. However, particularly due to issues pertaining to mud surfaces in Ethiopia, there were a greater number of instances where observed efficacy fell short of WHO range.

#### Pirimiphos-methyl (EC/CS)

Because of a notable difference in efficacy and performance, the two formulation types of pirimiphos-methyl were noted separately for each observation in Additional file 1: Table S1 and associated calculations of average efficacy were also performed separately. However, for brevity, all measures were included in Fig. 5 and Additional file 5: Table S5. Residual efficacy testing was performed in conjunction with spray campaigns that took place from 2010 to 2015. The residual efficacy (in months) of EC formulation lasted for 1.9 months (± 0.35) when tested on cement and mud surfaces, for a total of eight observations. For the CS formulation, the range of efficacy spanned from two to more than nine months. Cement, mud, mud covered with kaolin, wood, bamboo, dung, painted, and thatch surfaces were all included in bioassays performed in Benin, Ethiopia, Ghana, Liberia, Madagascar, Malawi, Mali, Senegal, Zimbabwe and Zambia. The average length of observed efficacy was 5.30 months (± 1.90). Of the 57 testing observations, there were 13 observed instances (23%) where the length of residual efficacy did not meet the minimum proposed in WHO guidelines (range of four to six months for CS formulation). Of these 13 observations, cement, mud and mud covered with kaolin surfaces were the only types represented. There were 21 instances where continued efficacy monitoring showed that pirimiphos-methyl consistently resulted in greater than 80% mosquito mortality for longer than six months; all of these observations were associated with the CS formulation.

### Direct comparative analysis

#### Country to country comparisons

Country-level comparisons of residual efficacy resulted in a large number of statistically significant differences in insecticide activity. For instance, the residual efficacy of bendiocarb applied to mud surfaces in Ethiopia was shorter than its residual efficacy on mud surfaces in Mali (*P* = 0.015; *n* = 2656 mosquitoes). Similarly, the residual efficacy of deltamethrin sprayed on cement surfaces in Rwanda was shorter than its residual efficacy on cement surfaces in Nigeria (*P* < 0.001; *n* = 13,530 mosquitoes) and Mozambique (*P* < 0.001; *n* = 11,520 mosquitoes). The bio-efficacy of deltamethrin sprayed on mud surfaces in Rwanda was shorter than in Mozambique (*P* = 0.009; *n* = 11,520 mosquitoes) and in Ethiopia (*P* < 0.001; *n* = 6000 mosquitoes). Pirimiphos-methyl applied to wood and cement surfaces in Liberia showed shorter residual efficacy than that reported on wood surfaces in Madagascar (*P* = 0.007; *n* = 4621 mosquitoes) and cement surfaces in Ghana (*P* < 0.001; *n* = 5666 mosquitoes). Additionally, pirimiphos-methyl sprayed on mud surfaces in Ghana performed longer than on mud surfaces in Ethiopia (*P* = 0.008; *n* = 6310 mosquitoes), Mali (*P* = 0.002; *n* = 5834 mosquitoes), Senegal (*P* < 0.001; *n* = 12,906 mosquitoes), and Zimbabwe (*P* = 0.002; *n* = 16,307 mosquitoes).

#### Insecticide type comparisons

In Ethiopia, the residual efficacy of bendiocarb was shorter than that of deltamethrin (*P* < 0.001; *n* = 1680 mosquitoes) and pirimiphos-methyl CS (*P* < 0.001; *n* = 4948 mosquitoes) on mud surfaces. Similarly, in Madagascar, pirimiphos-methyl CS lasted longer than bendiocarb on mud (*P* < 0.003; 4680 mosquitoes) and wood (*P* < 0.02; 6840 mosquitoes) surfaces. However, in Ghana no statistically significant difference was observed in the residual activity of pirimiphos-methyl compared to alpha-cypermethrin on cement (*P* = 0.97; *n* = 12,851 mosquitoes), mud (*P* = 0.91; *n* = 13,559 mosquitoes) and wood (*P* = 0.80; *n* = 9009 mosquitoes) surfaces. Lastly, bendiocarb residual efficacy in Rwanda was significantly different from that of deltamethrin on cement (*P* < 0.001, *n* = 23,280 mosquitoes) and mud (*P* = 0.014, *n* = 11,640 mosquitoes) surfaces.

#### Surface type comparisons

The residual activity of insecticides used for IRS presented statistically significant variation by surface type in some places but not in others. For instance, the residual efficacy of bendiocarb on mud surfaces in Ethiopia was significantly shorter than on painted (*P* < 0.001; *n* = 3497 mosquitoes) and dung (*P* < 0.001; *n* = 3179 mosquitoes) surfaces. In Ghana, alpha-cypermethrin lasted longer on mud surfaces than on cement surfaces (*P* = 0.04; *n* = 6680 mosquitoes). The residual activity of bendiocarb on mud surfaces coated with cement in Mali was significantly different from cement (*P* = 0.02; *n* = 999 mosquitoes) and mud (*P* = 0.045; *n* = 932 mosquitoes) surfaces. Additionally, the residual efficacy of bendiocarb in Rwanda was significantly different between mud and cement surfaces (*P* = 0.01; *n* = 17,970 mosquitoes). The residual activity of pirimiphos-methyl was similar on dung, painted and mud surfaces in Ethiopia; on mud, bamboo, thatched, and wood surfaces in Madagascar; and on mud and cement surfaces in Liberia, Senegal and Zambia. The performance of bendiocarb was also approximately similar on cement, wood and mud surfaces in Liberia; on mud and wood surfaces in Madagascar; and on cement and mud surfaces in Mali.

#### Mosquito source comparisons

Where mortality data were available, comparisons were made with respect to wild-caught *versus* colony-raised mosquitoes that were used in bioassays. The observed residual efficacy of pirimiphos-methyl in Ghana was significantly different when colony mosquitoes were used in assays as opposed to wild-caught mosquitoes (*P* < 0.001; *n* = 26,299 mosquitoes). However, no significant differences were seen in the mosquito test mortality between wild and susceptible colony on surfaces sprayed with pirimiphos-methyl in Ethiopia (*P* = 0.96; *n* = 9901 mosquitoes), Zambia (*P* = 0.61; *n* = 5156 mosquitoes) and Zimbabwe (*P* = 0.55; *n* = 3720 mosquitoes).

#### Year of spray program comparisons

The residual efficacy of specific insecticides observed over the course of several years was also reported to be significantly different from year to year. For instance, in Ethiopia, the residual efficacy of bendiocarb sprayed in the 2013 campaign was longer than the residual efficacy observed after the 2011 campaign (*P* < 0.001; *n* = 1860 mosquitoes) and 2015 campaign (*P* < 0.001; *n* = 3986 mosquitoes). Similarly, significantly different residual performance was observed between bendiocarb applications in Madagascar in 2012 and in 2013 (*P* = 0.01; *n* = 5040 mosquitoes). In Rwanda, though the residual efficacy of bendiocarb observed in the 2013 and 2014 spray campaigns was three months, there was a statistically significant difference between the years in terms of mosquito test mortality (*P* = 0.02; *n* = 8280 mosquitoes). Test mosquito mortality was higher at month four in 2014 than in 2013.

Deltamethrin also displayed significant differences in residual efficacy from year to year. In Rwanda, when sprayed on mud surfaces, the residual efficacy of deltamethrin was significantly different between 2013 and 2014 (*P* < 0.001; *n* = 6169 mosquitoes), and between 2012 and 2014 (*P* < 0.001, *n* = 6480 mosquitoes).

Overall, of the instances of statistically significant differences obtained when making comparisons based on country, insecticide, surface type, source mosquitoes, and year of spray application, there were a total of 49 significant comparisons. Of the 49 instances of statistical differences in length of residual efficacy, 24 significant comparisons were between countries, eight were from year to year, four were between types of insecticides, and two were between susceptible and wild mosquitoes in a given setting.

## Discussion

We have compiled the residual bio-efficacy of five IRS products from three classes of insecticides assessed under operational conditions of PMI/USAID-supported IRS programs in 17 countries. The summarized results indicate that residual efficacy had a wide variance across settings, years, and sprayable surfaces, and deviations from WHO residual efficacy ranges were detected for all of the insecticides covered in this study.

Additional evidence of wide variation in insecticide efficacy over time is available in the associated scientific literature. Consistent with results reported here, the residual efficacy of pyrethroids has been found to both exceed and fall short of thresholds suggested in WHO guidelines. For alpha-cypermethrin WP, previous studies have had mixed results, and showed residual efficacies shorter than the WHO’s report [41–43], in keeping with the report [43], or longer [41] depending on the location and surface sprayed. As seen in Fig. 1, there were no instances where reported residual efficacy of alpha-cypermethrin failed to reach the WHO lower limit, and it exceeded the WHO maximum threshold on four occasions. Similarly, for deltamethrin WG, previous studies have shown a residual efficacy in keeping with the WHO report [18], or longer [18, 44, 45]. One study, from Iran using a dosage of 25 mg/m^2^ against a laboratory-reared *Anopheles stephensi* strain, found the residual effectiveness shorter than three months on mud and cement surfaces [26]. Results from PMI-supported programs, as reported in Fig. 2, also demonstrate the large variation in residual efficacy, including instances of failing to reach the minimum WHO threshold and several observations that exceed the maximum WHO threshold.

Previous studies assessing the residual life of bendiocarb also produced mixed results, with some studies finding a residual life below that specified by WHO [21] or in keeping with WHO ranges [18, 46, 47]. In Benin in 2010, we observed seven months of residual efficacy, which may have been due to a progressive accumulation of insecticide particles on the wall surfaces as bendiocarb had been sprayed twice during the year. However, most of the differences in the residual life of bendiocarb we observed between countries are likely strongly influenced by surface type. In Benin, Djenontin et al. [21] found the residual life of bendiocarb to be between five and seven weeks on walls made of red clay (a mixture of sand and cement). Insecticides break down rapidly in an alkaline medium. Ordinary cement is highly alkaline, which may cause faster decomposition of insecticides, including carbamates, leading to short residual life, and may explain the short residual life of bendiocarb found in Mali in our data. On the other hand, in Rwanda, bendiocarb performed relatively better on cement as compared to mud surfaces (*P* = 0.01). Significant differences were not seen in the performance of bendiocarb between mud and wood surfaces in Madagascar, which is in line with a finding by Maharaj et al. [47].

There was a significant difference (*P* < 0.001) in the performance of bendiocarb between mud and dung walls in Ethiopia. Results from a recent study conducted using experimental huts in Adama District (East Central Ethiopia) by the PMI Africa Indoor Residual Spraying Project showed a strong association between the surface type and decay rate of bendiocarb [48]; the activity of bendiocarb declined faster on more porous mud surfaces, compared to smoother painted surfaces and dung surfaces. Outside of surface type, other factors that may have contributed to the shorter observed residual life in Mali and Ethiopia include porosity of the wall surfaces [18] and the amount of insecticide deposited on the surfaces [26].

Pirimiphos-methyl CS is a relatively new formulation of insecticide, added to the list of IRS insecticides recommended by WHOPES in 2013. A recent study has shown that temperature before, during, and after exposure can influence how well organophosphate and pyrethroid insecticides kill mosquitoes [49]. Although the effect of temperature as reported by the authors was on the susceptibility of vectors to insecticides [49], the same principle might apply for cone bioassay tests. The influence of temperature on mosquito mortality in cone bioassays is likely to be complex since the tests are done in uncontrolled, natural field conditions where variations can occur during the exposure period. Furthermore, studies by Hadaway & Barlow [27] have shown that the sorption of organophosphate and carbamate insecticides on dried muds is influenced by atmospheric humidity and that the biological activity of a given concentration of absorbed insecticide increases with increasing humidity. The study demonstrated that an increase to high humidity for about 24 h can result in sufficient migration of insecticide to the surface and increase bioavailability to the mosquito and increase mortality. Potential variations in humidity by area and season might have contributed to variations of the residual life of insecticides reported in this study.

A small-scale trial of pirimiphos-methyl CS conducted in Benin showed an effective residual life of nine months, despite being sprayed at a lower dosage of 0.5 g/m^2^ [50]. However, a shorter than nine-month residual life was observed from our tests in Benin, Liberia, Mali, Madagascar, Senegal, Zambia and Zimbabwe. Oxborough et al. [51] reported five and seven months of residual activity of pirimiphos-methyl CS on mud and concrete surfaces, respectively, from a study conducted in experimental huts in Tanzania. In another experimental hut study, conducted in Côte d’Ivoire [11], the residual efficacy of pirimiphos-methyl CS was found to be four and five months on mud and concrete surfaces, respectively. Haji et al. [52] reported eight months of residual effect of pirimiphos-methyl CS on water paint, cement plastered, and mud and lime wash surfaces. They observed longer than nine months of bio-efficacy on oil paint surface. During our observations, residual efficacy of a shorter duration than WHO specifies was noted in Benin, Liberia, Mali, Senegal and Zimbabwe. The variations might have been due to differences in environmental conditions, surface type including variations in mud composition, quality of spraying, and human interference on the sprayed surfaces.

Variable results were observed in the residual activity of insecticides when age standardized wild and susceptible colonies of mosquitoes were simultaneously exposed to same sprayed surfaces. In Ghana the residual efficacy of pirimiphos-methyl CS sprayed on cement and mud surfaces was shorter by at least a month when measured using wild mosquitoes compared with a susceptible colony. The difference was statistically significant. The wild mosquitoes used for the test were susceptible to pirimiphos-methyl, and the cone test mortality rate was high and similar for wild and susceptible mosquito colonies for a few months after spraying though results diverged over time. The trend was consistent in 2013, 2014 and 2015. The wild mosquitoes in Ghana might have developed some level of tolerance to the insecticide that was not detectable using diagnostic dosages but started to survive as the concentration of insecticide on sprayed surfaces declined due to insecticide bio-degradation. Recent reports of emergence of vector resistance to pirimiphos-methyl (unpublished data) in the country might be another good indication that an undetectable level of resistance gene has been circulating in *An. gambiae* (*s.l.*) mosquito populations in Ghana. In other countries, such as Zambia and Zimbabwe where there is no sign of emergence of vector resistance to pirimiphos-methyl, no difference in the residual efficacy was observed when measured using wild and susceptible mosquito colonies (Additional file 6: Table S6).

It is important to note that the average length of efficacy reported for each insecticide monitored through this activity is roughly within WHO guidelines; however, 71% of the alpha-cypermethrin, 48% of deltamethrin, 21% of bendiocarb, and 11% of pirimiphos-methyl bio-efficacy monitoring points were cut off before the residual activity of insecticides dropped below the 80% mortality threshold. Therefore, the average residual lives of each insecticide reported here are likely conservative estimates.

Significant variations in the residual life of insecticides were also noted among countries. Over all the residual activity of insecticides seems to last longer in some countries as compared to others. For example, pirimiphos-methyl lasted longer in Ghana compared to most other PMI AIRS supported countries such as Benin, Ethiopia, Liberia, Mali, Senegal and Zimbabwe. Deltamethrin lasted longer in Rwanda when compared with Ethiopia, Nigeria and Mozambique and bendiocarb lasted longer in Rwanda and Uganda as compared to Mali, Benin, Senegal and Ethiopia. The wall surfaces of sprayed houses in Ghana and Rwanda seem to be smoother and less porous than other countries compared. This might be one of the most likely reasons why better performance of insecticides was observed in these two countries. Longer residual activity of the carbamate (bendiocarb), pyrethroid (deltamethrin) and organophosphate (pirmiphos-methyl) were observed in Madagascar when compared with countries like Mali, Ethiopia and Benin. Most of the wall surfaces in Madagascar are either made of wood or falafa (Bana leaf). The rate of bio-degradation of insecticides is known to be slow on wood and related surfaces compared with mud and cement surfaces. Other environmental factors and human activities might also have contributed to the difference in residual activity of insecticides. Further study is needed to tease out factor/s involved in the variations observed.

One of the limitations of this study is the absence of data on the amount of insecticide deposited on the wall surfaces. Although all efforts were made to ensure insecticide was mixed and sprayed at WHO-recommended doses, the initial concentration of insecticides sprayed on the walls may have varied by country and time, possibly explaining some of the variations observed in residual activity. The WHO cone bioassay test was used as a proxy indicator of the spray quality. The results from the initial bioassay tests conducted at the beginning of the spray campaigns indicated high test mosquito mortality rates (with negligible control mosquito mortality). Though obtaining high test mortality is a good indicator, it is not sufficient to help us conclude that the quality of the spraying was high with reasonable confidence without measuring the amount of insecticide deposited. The same houses at initial testing were followed for all residual life data collections. Furthermore, differences in initial concentration may be expected in large-scale IRS projects, and monitoring of residual life may help detect and correct issues with implementation of IRS. In addition to cone bioassay testing, we recommend simultaneous use of the filter paper method to assess spray quality. This method will enable countries to directly estimate the amount of insecticide deposited on the sprayed surfaces through the chemical residue analysis of the filter papers using high-performance liquid chromatography. Data from the two methods can then be triangulated to better inform the program.

Secondly, when wild mosquitoes were used for the cone bioassay tests, molecular identification by sibling species was not done. In countries where difference in residual life of insecticides varied between wild and susceptible colony mosquitoes were observed potentially due to undetectable level of resistance and more than one sibling species of *An. gambiae* (*s.l.*) co-exist with difference in their response to insecticides, the difference in species composition might have contributed to the difference in residual life of insecticides when compared among different countries.

Finally, in Ghana all the three WHO cones used to measure the quality of spraying and residual activity of insecticides were placed in parallel at 1.0 m above the floor when bioassay tests were done unlike all the other countries where the cones were placed at three different heights (0.5 m, 1.0 m and 1.5 m). This difference in heights might have contributed to the difference in residual life of insecticides observed between Ghana and other countries though comparison in test mortality and residual life among the three heights in other countries like Senegal, Liberia, Mali and Zimbabwe where there was found to be a statistically significant difference with Ghana did not produce significant differences (data not shown).

## Conclusions

Data on the effective residual life of insecticides are critical to determine the frequency and timing of IRS operations in order to adequately protect populations at risk of malaria during high transmission seasons. The insecticide residual efficacy results presented here show that routine program monitoring provides essential information that may not be captured in controlled studies but is vital to guiding IRS operations. For example, bendiocarb presented shorter residual life than the malaria transmission season in Mali, which is about four months. Based on these data, PMI, in collaboration with the government of Mali, switched the choice of insecticide for IRS from bendiocarb to pirimiphos-methyl CS. This decision was made to ensure populations were fully protected for the high malaria transmission season. The effect of bendiocarb on *Anopheles* mortality showed wide variation in different sites within countries and between countries. With deltamethrin WG and alpha-cypermethrin WP, data from this assessment revealed on average higher residual life of these insecticides as compared to results reported by other authors. Pirimiphos-methyl CS showed longer residual efficacy in some countries and shorter in others than in previously published studies. These results underscore the need to collect local residual life data when introducing insecticides to new areas for IRS.

## Additional files

Additional file 1: Table S1. Summary of insecticide residual bio-efficacy monitoring sites by year, insecticide, and surface type 2008–2015. (XLSX 13 kb)

Additional file 2: Table S2. Summary of monthly number exposed and percent mortality of insectary reared susceptible colony of *Anopheles gambiae* and *Anopheles arabiensis* after WHO standard cone bioassay tests on alpha-cypermethrin sprayed surfaces by country and surface type, 2008–2012. (XLSX 48 kb)

Additional file 3: Table S3. Summary of monthly number exposed and percent mortality of insectary reared susceptible colony of *Anopheles gambiae* and *Anopheles arabiensis* and wild *Anopheles gambiae* (*s.l.*) after WHO standard cone bioassay tests on deltamethrin sprayed surfaces by country, mosquito source and surface type, 2010–2015. (XLSX 23 kb)

Additional file 4: Table S4. Summary of monthly number exposed and percent mortality of insectary reared susceptible colony of *Anopheles gambiae* and *Anopheles arabiensis* and wild *Anopheles gambiae* (*s.l.*) after WHO standard cone bioassay tests on bendiocarb sprayed surfaces by country, mosquito source and surface type, 2010–2015. (XLSX 71 kb)

Additional file 5: Table S5. Summary of monthly number exposed and percent mortality of insectary reared susceptible colony of *Anopheles gambiae* and *Anopheles arabiensis* and wild *Anopheles gambiae* (*s.l.*) after WHO standard cone bioassay tests on pirimiphos-methyl sprayed surfaces by country, mosquito source and surface type, 2010–2015. (XLSX 29 kb)

Additional file 6: Table S6. Detailed statistical analysis results. (XLSX 25 kb)

## Abbreviations

CS: capsule suspension
DDT: dichlorodiphenyltrichloroethane
EC: emulsion concentrate
IRS: indoor residual spraying
PMI: United States President’s Malaria Initiative
SC: suspension concentrate
USAID: United States Agency for International Development
WG: wettable granule
WHO: World Health Organization
WHOPES: World Health Organization Pesticide Evaluation Scheme
WP: wettable powder
